# Obesity and poor breast cancer prognosis: an illusion because of hormone replacement therapy?

**DOI:** 10.1038/sj.bjc.6605025

**Published:** 2009-04-14

**Authors:** L Rosenberg, K Czene, P Hall

**Affiliations:** 1 Department of Medical Epidemiology and Biostatistics, Karolinska Institute, Box 281, SE-171 77, Stockholm, Sweden

**Keywords:** body mass index, breast neoplasm, oestrogen replacement therapy, mammography, prognosis

## Abstract

High body mass index (BMI) and use of hormone replacement therapy (HRT) increase the risk of postmenopausal breast cancer. It has been shown that BMI modifies the effect of HRT, as its influence is most pronounced in lean women. We investigated the influence of BMI and HRT on prognosis in 2640 postmenopausal women diagnosed with breast cancer in Sweden in 1993–1995, taking into account HRT and mammography before diagnosis. Logistic and Cox regression were used. In non-users of HRT, obese women (BMI >30) compared with normal weight women (BMI <25) had a similar prognosis (hazard ratio (HR) 1.1, 95% confidence interval (CI) 0.8–1.6), despite larger tumours found in obese women. Obese HRT users had less favourable tumour characteristics and poorer prognosis compared with normal weight women (HR 3.7, 95% CI 1.9–7.2). The influence of BMI on breast cancer prognosis was similar whether diagnosed by mammographic screening or not. We found a similar prognosis of postmenopausal breast cancer-specific death regardless of BMI in non-users of HRT, but among HRT users obesity was associated with a poorer breast cancer prognosis.

Obesity has repeatedly been associated with breast tumours of larger size, lymph node positivity, and poor prognosis (Chlebowski *et al*, 2002; Dal Maso *et al*, 2008), although some studies have produced contrasting findings (Dignam *et al*, 2003; Berclaz *et al*, 2004). However, many studies lack information on the use of hormone replacement therapy (HRT) and mammography examinations before diagnosis. Use of HRT interacts, or rather competes, with obesity on breast cancer risk among postmenopausal women, probably by sharing hormonal carcinogenic pathways (van den Brandt *et al*, 2000; Morimoto *et al*, 2002). The HRT effect on risk is most pronounced in lean postmenopausal women, in whom the endogenous production of oestrogen is the lowest. Obese women might benefit more from mammographic screening than normal weight women because of larger breasts, more difficult to palpate, and a lower mammographic breast density, and thereby a higher sensitivity of the mammography (Porter *et al*, 2006; Kerlikowske *et al*, 2008). Therefore, not taking HRT use and mammographic screening pattern into consideration could create spurious relationships between prognosis and obesity. Obesity is linked to an increased all-cause mortality (Lahmann *et al*, 2002; Adams *et al*, 2006), which means that using all-cause mortality as the outcome might obscure the association between obesity and breast cancer death. We therefore studied the influence of obesity on tumour characteristics and cause-specific mortality in postmenopausal breast cancer patients, taking into account HRT use before diagnosis and mode of detection.

## Materials and methods

We used information from all cases initially participating in a population-based case-control study conducted in Sweden between October 1993 and March 1995. Detailed information on data collection is given elsewhere (Magnusson *et al*, 1999). Briefly, all women born in Sweden aged 50–74 years at first diagnosis of invasive breast cancer were eligible. The investigation was approved by all the seven regional ethical review boards in Sweden, and informed consent was obtained from each participant. The mean interval from diagnosis to answering the mailed questionnaire was 4.3 months.

Out of 3979 women with a first diagnosis of breast cancer, 3345 women (84%) participated in the study. Menopause was defined as the age at last menstrual period or age at bilateral oophorectomy, if 1 year or more before data collection. Exclusions were made because of earlier cancer (150 cases), noninvasive breast cancer according to patient record not reported from the regional cancer registry (58), diagnosed outside study period (19), lack of permission to access patient record information (58), other cancer than breast cancer (1), being premenopausal (198) as well as being below age 55 years with unknown age at menopause (possibility of being premenopausal, 202 cases), missing age at first birth (5), and missing height or recent weight (14). Thus, 2640 cases remained for analysis. Data on possible breast cancer risk factors including information on use of HRT were collected through a mailed questionnaire.

We collected information on mode of detection (screening or not), primary treatment (surgery, chemotherapy, radiotherapy, and endocrine therapy), and tumour characteristics (tumour size, lymph node involvement, grade, and oestrogen and progesterone receptor status) from patient records primarily from surgical and oncological units throughout Sweden. Information on grade was not in routine clinical use in Sweden at the time of the study, and is consequently missing in 33% of the cases. From breast radiology units, we collected information on date and reason for the mammographies (screening or referral) carried out before diagnosis.

We used the Swedish National Registration Number, unique to each citizen, to collect information on emigrations from the Swedish National Population Register, and to obtain information on date and cause of death until December 31 2003 from the Swedish Cause of Death Register. The latter register covers all residents, and has been shown to correctly classify 98% of deaths in breast cancer patients (Nystrom *et al*, 1995).

According to the World Health Organization (WHO), BMI (body mass index 1 year before answering the questionnaire, kg m^−2^) was classified into underweight (<18.5), normal weight (18.5–25), overweight (25–30), and obese (>30). As only 34 women were underweight, they were merged with the normal weight group. Recent mammography was defined as the mammography within 2 years and 2 months before diagnosis (yes/no), to cover the normal 2-year interval of mammographic screening and 2 months delay. Detection by screening was defined as diagnosis by the official screening programme or by other health checks without symptoms.

HRT was classified into ever use of (a) oestrogen in combination with progestin (oestrogen-progestin), (b) oestrogen alone, or (c) never use of HRT. Current oestrogen–progestin use was defined as the last use within 6 months before diagnosis. Progestin alone was largely classified as past use more than 10 years before diagnosis, and therefore not analysed, but excluded from never use of HRT. Oral estriol and local oestrogen treatment was not defined as HRT.

Grade was collected from pathological reports, and was classified according to the Nottingham histological grade or the Bloom-Richardson scale (Bloom and Richardson, 1957; Elston and Ellis, 1991). Oestrogen- or progesterone-positive was defined as ⩾0.05 fmol receptor per microgram DNA or ⩾10 fmol receptor per miligram protein.

### Statistical analyses

Means and frequencies of various background factors according to BMI groups were calculated, and *t*-test (for means) and *χ*^2^-tests (for frequencies) were carried out to detect differences between obese and normal weight women.

We used frequencies with *χ*^2^-tests, as well as polytomous multiple logistic regression to estimate odds ratios (OR) with 95% confidence intervals (CIs) for the associations between BMI groups and tumour characteristics, with one class of each tumour characteristic as control group and the other(s) as outcome(s). Age at diagnosis, parity, at first birth, menopause, current alcohol intake, socioeconomic status, and current smoking status were evaluated separately as potential confounders of the associations of interest, and were included in the final models if they affected any of the estimates more than 10%. In addition, we carried out the same analyses stratified by use of HRT.

We used the Kaplan–Meier method with log-rank test for assessing breast cancer-specific mortality distributions in relation to BMI among all women and stratified for HRT use. We estimated breast cancer-specific mortality rates, and used the Cox proportional hazards model to compare the HRs of such deaths between BMI categories, among all women and in strata by HRT use and by detection by screening. Heterogeneity of the BMI effect by HRT use and by detection by screening was tested using Wald *χ*^2^-test. Potential confounders were tested by inclusion in an age-stratified Cox model. Tumour characteristics (tumour size and lymph node involvement) and treatment are intermediates between BMI exposure and breast cancer survival, and were thus included in Cox models to assess the extent to which they could explain the observed associations.

## Results

Obese, compared with normal weight women, were older at diagnosis, drank less alcohol, were of lower socioeconomic status, were less often HRT users or current smokers, and were more often diagnosed by mammographic screening (Table 1). There were no differences between BMI groups regarding parity, age at first birth, number of children, or age at menopause. The proportion of women with a recent mammography (within 2 years and 2 months before diagnosis) did not differ between normal weight and obese women.

Compared with normal weight women, obese women more often had large tumours, lymph node positivity, and progesterone receptor-positive tumours (Table 2). We found no difference in grade or oestrogen receptor status between normal weight and obese women. Among never users of HRT there was a slight attenuation of the associations between obesity and both large tumour size and lymph node involvement (Table 2). On the other hand, the association between obesity and progesterone receptor positive-tumours was strengthened. In contrast, obese women ever using oestrogen–progestin therapy seemed to have tumours with less favourable prognostic characteristics. Obese compared with normal weight women seemed to more often have high-grade tumours, and there was a strong association with lymph node involvement, whereas there was no association with progesterone receptor status (Table 2). Women with oestrogen alone use were few, but as the patterns of tumour characteristics over BMI categories were similar compared with women with no HRT use (data not shown), oestrogen alone use was not further analysed.

**Figure 1:**
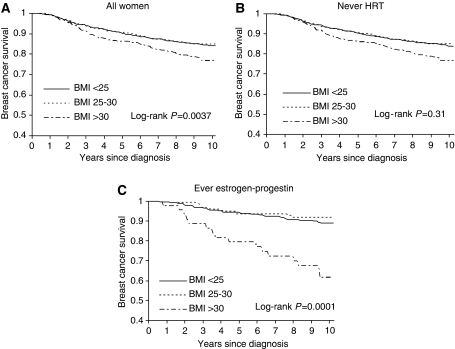
Kaplan–Meier curve of breast cancer-specific survival by body mass index. The Swedish Breast Cancer Study 1993–2003. (**A**) All women. (**B**) Never use of HRT. (**C**) Ever use of estrogen–progestin.

Among obese women, 35% died during the follow-up (median follow-up 9.5 years, range 8.8–10.2), whereas 24% of both overweight and normal weight women died (Table 1). Death because of breast cancer was more common than death because of other causes regardless of BMI. Breast cancer-specific survival was poorer among obese women compared with normal and overweight women (Figure 1A). BMI did not influence survival in those women not using HRT (Figure 1B), whereas among the ever oestrogen–progestin users, obese women had a poorer survival (Figure 1C).

Overall, obese compared with normal weight women had an increased risk of breast cancer death (HR 1.4, 95% CI 1.1–1.9; Table 3). When we stratified by the use of HRT, we found no association between BMI and survival among never HRT users (HR 1.1, 95% CI 0.8–1.6; Table 3), whereas this association was clearly evident among oestrogen–progestin users when comparing obese with normal weight women (HR 3.7, 95% CI 1.9–7.2). In these analyses, evidence of heterogeneity was found for use of HRT (*P*=0.0029). On the other hand, the influence of BMI on survival was similar whether diagnosed by mammography screening or not (*P*=0.98).

Adding tumour size and lymph node positivity to the models seemed to explain part of the increased risk of death for obese women (Table 3), but obese women ever using oestrogen–progestin still had twice the risk of dying of breast cancer compared with normal weight women (HR 2.3, 95% CI 1.1–5.2; Table 3). Primary treatments of the tumour were not related to BMI (Table 1), and, as expected, did not influence the survival estimates (data not shown). Among ever oestrogen–progestin users, 85% of normal weight women and 63% of obese women were current users. When the analysis was restricted to current oestrogen–progestin users, the results of a poorer survival among obese women were similar and significant (data not shown).

## Discussion

We found that among women not using HRT, obese compared with normal weight women more often had large and progesterone receptor-positive tumours, but similar breast cancer-specific survival. On the other hand, obese women using oestrogen–progestin had worse tumour characteristics and prognosis compared with normal weight women.

Our findings of modest associations between obesity and poor tumour characteristics and no association with breast cancer-specific survival in never users of HRT contradict most earlier studies (Chlebowski *et al*, 2002; Dal Maso *et al*, 2008). Use of HRT became increasingly popular during the 1990s, but dropped substantially after the report of adverse effects from the Women's Health Initiative. Studies conducted on populations diagnosed with breast cancer before 2002 often have a large proportion of current HRT users. As indicated by our data, not taking HRT use into consideration could introduce spurious associations when analysing the influence of BMI on prognosis. However, two large and well-designed studies have found similar breast cancer survival regardless of BMI without taking HRT use into consideration (Dignam *et al*, 2003; Berclaz *et al*, 2004).

Whether use of HRT before diagnosis is linked to a better breast cancer prognosis is not clear. Most observational studies indicate such an association, whereas the Women's Health Study randomized trial found HRT oestrogen–progestin use to be associated with larger and lymph node-positive tumours (Antoine *et al*, 2004). If HRT is linked to less aggressive tumours, our finding of a poorer survival among obese women only among HRT users could be explained as follows: the beneficial prognostic effect seen by HRT in most observational studies is confined to lean women, in whom the HRT effect is most pronounced. This beneficial effect of HRT is not seen in obese women.

The aim of studying factors that influence survival is to identify groups with a particularly bad prognosis, with the ultimate goal of individualising cancer treatment. Many studies of obesity and breast cancer have used overall death, which is inappropriate when studying the effect of certain exposures on breast cancer survival. Obese women are known to have a higher general mortality, mainly because of cardiovascular causes (Lahmann *et al*, 2002; Adams *et al*, 2006). Among young breast cancer women, in whom the vast majority of deaths are because of cancer, overall death can be a good proxy, but for postmenopausal women, the influence from other deaths are considerable and the association between obesity and breast cancer-specific survival from such studies is obscured. Therefore, despite the large number of earlier studies, we consider that more studies assessing breast cancer-specific survival are warranted. Another consideration is the method of diagnosis. Obese compared with normal weight women, have larger breasts, which makes palpation a less likely means of detection as reflected in large non-screening detected tumours diagnosed in obese women (Hunt and Sickles, 2000; Porter *et al*, 2006). On the other hand, obesity is related to a lower mammographic breast density (Hunt and Sickles, 2000; Boyd *et al*, 2006), thereby might give an earlier diagnosis. Thus, the association between obesity and breast cancer characteristics and survival might depend on screening intensity. We found that obese women not diagnosed by screening had larger tumours compared with normal weight women, but we could not see this transferred to poorer survival among obese compared with normal weight women.

Mammographic screening intensity influences the distribution of the breast cancer characteristics and survival by earlier cancer detection (Shen *et al*, 2005). If screening use differs between obese and non-obese women, this might introduce a bias, neglected in most earlier studies. In our study, as in another (Porter *et al*, 2006), we did not find obesity to influence screening intensity.

Our findings of a poorer survival among obese compared with normal weight HRT users are statistically significant, and therefore possibility of chance is small. However, as the study is observational, other factors may be responsible, such as better health, less patient's or doctor's delay, and possibly better treatment among normal weight women. Treatment was considered, and was similarly distributed among BMI groups.

Our main conclusion is that obesity does not seem to confer a poor breast cancer-specific survival in never HRT users. The worsened prognosis seen in postmenopausal obese breast cancer patients is confined to HRT users. Tumours in normal weight HRT users showed a less aggressive behaviour. The influence of BMI on breast cancer survival was not explained by mode of detection.

## Figures and Tables

**Table 1:** Baseline characteristics of 2640 postmenopausal Swedish women diagnosed with breast cancer in 1993–1995, by body mass index

	**BMI**			
	**<25**	**25–30**	**>30**	***P*-value**
No. of cases	1267	997	376	
				
*Mean*				
Age at diagnosis (years)	62.4	64.0	64.7	<0.0001
Parity	1.8	1.9	1.8	0.34
Age at first birth (years)	25.5	25.3	25.3	0.68
Age at menopause^a^ (years)	50.5	50.7	50.4	0.88
Current alcohol intake, g day^−1^	2.9	2.3	1.7	<0.0001
				
*Frequency* (%)				
High socioeconomic status^b^	54	47	39	<0.0001
*Ever HRT*				
Ever oestrogen alone	14	10	7	0.0005
Ever oestrogen–progestin	30	15	12	<0.0001
Current smoker	30	18	13	<0.0001
Recent mammography^c^	65	60	64	0.64
Detection by screening	57	63	65	0.012
*Primary treatment*				
Surgery	99	100	99	0.86
Chemotherapy	8	9	11	0.15
Antioestrogenic therapy	54	51	57	0.30
Radiotherapy	64	62	63	0.96
Deceased at end of follow-up	24	24	35	0.0003
*Cause of death*				
Breast cancer	15	15	21	0.0008
Other cause	9	9	14	0.0014

BMI=body mass index (kg m^−2^); HRT=hormone replacement therapy.

*P-*value: two sided *t*-test for means and *χ*^2^-test for the frequencies comparing BMI >30 *vs* BMI <25.

a Among women with a natural menopause.

b High level includes medium- and high-level white collar workers.

c Mammography within 2 years and 2 months before diagnosis.

**Table 2:** Distribution of tumour characteristics by body mass index, and adjusted odds ratios for tumour characteristics in relation to body mass index, by the use of HRT: the Swedish Breast Cancer Study 1993–2003

	**BMI**				**Adjusted OR**			
**Tumour characteristics**	**<25**	**25–30**	**>30**	*P*-value*	**25–30**	**95% CI**	**>30**	**95% CI**
*All women*								
*Tumour size (cm)*								
⩽2	919 (73)	702 (71)	241 (64)					
>2	333 (27)	287 (29)	133 (36)	0.0014	1.2	1.0, 1.4	1.6	1.2, 2.0
*Lymph node positivity*								
No	854 (70)	651 (68)	229 (64)					
Yes	370 (30)	313 (32)	127 (36)	0.046	1.1	0.9, 1.4	1.3	1.0, 1.7
*Grade*								
1	129(16)	104 (15)	37 (15)					
2	359 (43)	272 (40)	104 (41)		0.9	0.6, 1.2	0.9	0.6, 1.4
3	339 (41)	309 (45)	110 (44)	0.29	1.1	0.8, 1.5	1.1	0.7, 1.6
*Oestrogen receptor*								
Positive	710 (78)	540 (77)	229 (81)					
Negative	199 (22)	158 (23)	53 (19)	0.44	1.1	0.9, 1.4	0.9	0.6, 1.3
*Progesterone receptor*								
Positive	570 (64)	468 (68)	198 (71)					
Negative	316 (36)	220 (32)	82 (29)	0.029	0.9	0.7, 1.1	0.8	0.6, 1.0
								
*Never hormone replacement therapy*								
*Tumour size (cm)*								
⩽2	531 (72)	519 (71)	186 (64)					
>2	207 (28)	213 (29)	103 (36)	0.033	1.1	0.9, 1.4	1.4	1.1, 1.9
*Lymph node positivity*								
No	496 (69)	483 (68)	179 (66)					
Yes	222 (31)	229 (32)	93 (34)	0.33	1.1	0.9, 1.3	1.2	0.9, 1.6
*Grade*								
1	67 (13)	65 (13)	31 (16)					
2	229 (46)	207 (41)	83 (43)	0.90	0.8	0.6, 1.3	0.7	0.4, 1.1
3	204 (41)	232 (46)	80 (41)		1.1	0.8, 1.7	0.8	0.5, 1.3
*Oestrogen receptor*								
Positive	421 (79)	395 (77)	174 (81)					
Negative	113 (21)	116 (23)	41 (19)	0.73	1.1	0.8, 1.5	0.9	0.6, 1.3
*Progesterone receptor*								
Positive	316 (61)	345 (69)	150 (70)					
Negative	204 (39)	158 (31)	63 (30)	0.0036	0.7	0.5, 0.9	0.6	0.4, 0.9
								
*Ever oestrogen–progestin therapy*								
*Tumour size (cm)*								
⩽2	278 (76)	110 (73)	28 (65)					
>2	90 (24)	41 (27)	15 (35)	0.15	1.2	0.8, 1.9	1.9	0.9, 3.8
*Lymph node positivity*								
No	260 (71)	97 (66)	21 (50)					
Yes	104 (29)	51 (35)	21 (50)	0.0051	1.3	0.9, 2.0	2.7	1.4, 5.2
*Grade*								
1	54 (23)	26 (24)	3 (10)					
2	92 (40)	36 (34)	10 (34)		0.7	0.4, 1.4	2.0	0.5, 7.7
3	86 (37)	45 (42)	16 (55)	0.084	1.3	0.7, 2.4	3.4	0.9, 12.7
*Oestrogen receptor*								
Positive	207 (78)	86 (79)	28 (78)					
Negative	60 (22)	23 (21)	8 (22)	0.86	1.1	0.6, 1.9	1.2	0.5, 3.0
*Progesterone receptor*								
Positive	187 (71)	73 (68)	24 (67)					
Negative	75 (29)	34 (32)	12 (33)	0.45	1.3	0.8, 2.2	1.7	0.8, 3.7

BMI=body mass index (kg m^−2^); CI=confidence interval; HRT=hormone replacement therapy; OR=odds ratio.

^*^P-value: Two-sided Mantel–Haenszel *χ*^2^ for the distribution between groups.OR adjusted for age at diagnosis (5-year categories), parity (0, 1, 2, 3, and >3), current smoking (yes/no), and age at menopause (<45, 45–49, 50–54, and ⩾55 years).

**Table 3:** Breast cancer-specific mortality in relation to body mass index before diagnosis, by the use of HRT or by detection mode: the Swedish Breast Cancer Study 1993–2003

	**Breast cancer deaths/exposed**	**Breast cancer mortality rate^a^**	**HR^b^**	**95% CI**	**HR^c^**	**95% CI**
*All women*						
*BMI*						
<25	162/1090	1.8	1.0	Ref.	1.0	Ref.
25–30	126/873	1.7	1.0	0.8, 1.2	0.9	0.7, 1.2
>30	66/322	2.6	1.4	1.1, 1.9	1.2	0.9, 1.6
						
*Never hormone replacement therapy*						
*BMI*						
<25	108/628	2.1	1.0	Ref.	1.0	Ref.
25–30	99/645	1.9	0.9	0.7, 1.2	0.8	0.6, 1.1
>30	45/243	2.3	1.1	0.8, 1.6	0.9	0.6, 1.3
						
*Ever oestrogen–progestin therapy*						
*BMI*						
<25	36/329	1.2	1.0	Ref.	1.0	Ref.
25–30	10/130	0.9	0.8	0.4, 1.6	1.0	0.5, 2.1
>30	13/40	4.3	3.7	1.9, 7.2	2.3	1.1, 5.2
						
*Detected by mammographic screening*						
*BMI*						
<25	56/612	1.0	1.0	Ref.	1.0	Ref.
25–30	52/548	1.1	1.0	0.7, 1.5	0.9	0.6, 1.4
>30	29/209	1.7	1.5	0.9, 2.3	1.0	0.6, 1.6
						
*Not detected by mammographic screening*						
*BMI*						
<25	104/466	2.8	1.0	Ref.	1.0	Ref.
25–30	74/321	2.9	1.1	0.8, 1.5	0.9	0.7, 1.3
>30	36/112	4.6	1.6	1.1, 2.4	1.4	0.9, 2.1

BMI=body mass index (kg m^−2^); CI=confidence interval; HR=hazard ratio; HRT=hormone replacement therapy.

a Breast cancer deaths per 100 person-years.

b Adjusted for age at diagnosis in 5-year categories, and current alcohol intake (0, 0–4.9, 5–9.9, and ⩾10 g day^−1^).

c Adjusted for age at diagnosis, current alcohol intake (0, 0–4.9, 5–9.9, and ⩾10 g day^−1^), tumour size (⩽2, >2 cm), and lymph node positivity (no, yes).
